# Screening of Stable Reference Genes in *Solea senegalensis* Juveniles for Nervous Necrosis Virus (NNV) Vaccination Assays

**DOI:** 10.3390/vetsci13080790

**Published:** 2026-08-08

**Authors:** Lucía Vázquez-Salgado, Carmen López-Vázquez, Isabel Bandín, Sandra Souto

**Affiliations:** Aquatic One Health Research Center (ARCUS), Universidade de Santiago de Compostela (USC), 15782 Santiago de Compostela, Spain; mdelcarmen.lopez.vazquez@usc.es (C.L.-V.); isabel.bandin@usc.es (I.B.)

**Keywords:** reference gene, gene expression, normalisation, *rps4*, *ubq*, RT-qPCR

## Abstract

Quantifying the expression of immune-related genes is essential for evaluating vaccine efficacy. However, obtaining reliable RT-qPCR results requires the use of stable reference genes. In this study, the expression stability of eight candidate reference genes (*actb1*, *actb2*, *eef1a*, *gapdh2*, *hprt1*, *ubq*, *18S*, and *rps4*) was evaluated in brain, kidney, and gut tissues from naïve and virus-infected juvenile sole. Gene stability was assessed through two statistical algorithms (geNorm and NormFinder) and the integrative tool RefFinder, and the results were integrated through RankAggreg for a robust consensus ranking. Among the tested genes, *rps4* and *ubq* showed the highest stability across tissues and experimental conditions and were sufficient for accurate normalisation. The suitability of these genes was validated in vaccinated sole for normalisation of the expression of proinflammatory cytokines (*il1β*, *il6*, *tnfα*), supporting their recommendation as optimal reference genes. Conversely, *actb1* and *hprt1* were the least stable genes and compromised the reliable quantification of proinflammatory cytokines in vaccinated fish.

## Simple Summary

Gene expression analysis by RT-qPCR is widely used to evaluate the immune response induced by vaccination, but its accuracy depends on the use of appropriate reference genes. In this study, we identified the most stable reference genes for gene expression analysis in juvenile Senegalese sole and confirmed their suitability in vaccinated fish. Our results also demonstrate that the use of unsuitable reference genes may lead to misleading conclusions. These findings provide a robust tool for future vaccine studies and demonstrate the importance of selecting appropriate reference genes to obtain reliable and biologically meaningful results.

## 1. Introduction

Senegalese sole (*Solea senegalensis*) is a marine flatfish species of high commercial value in aquaculture. Production of this species is severely jeopardised by the Nervous Necrosis Virus (NNV) and to date, there are no available vaccines that protect sole against the infection [1]. The development of an effective vaccine requires an appropriate assessment of the immune response elicited, including accurate quantification of the expression of immune-related genes. However, unlike more extensively studied species, such as zebrafish and other important farmed species including salmonids, groupers, or sea bass, this species lacks standardised commercial tools for immunological research, with only limited information and scarce non-customised resources available. RT-qPCR is an efficient method to quantify the transcriptional expression of genes, but accurate quantification depends on several variables such as sample quality and the efficiency of enzymatic reactions, among others. To counteract biological and technical variability, the expression level of a target gene must be compared to that of a reference gene subjected to the same treatment [2,3,4]. A suitable reference gene must have a stable expression in different tissues and experimental conditions, allowing unequivocal detection of differences in target gene expression regardless of the experimental setup [2,5,6,7,8].

The genes most commonly used as references in transcriptomic studies are those encoding β-actin, glyceraldehyde-3-phosphate dehydrogenase (GAPDH), albumin, and/or ribosomal proteins [9]. However, several studies have shown that the expression of these genes depends on tissue type and experimental design [3,6,10,11,12,13]. In addition, according to the MIQE guidelines [14], there is no universally ideal reference gene, and studies based on a single gene may lead to an incorrect normalisation factor [11]. Therefore, an appropriate set of reference genes for accurate and reliable normalisation should be experimentally determined prior to assessing the expression of the gene of interest [8,15]. To evaluate the suitability of a panel of candidate genes for normalisation, several computational tools have been developed to rank gene stability using statistical algorithms [2,8,9].

The aim of this study was to determine an appropriate set of reference genes in Senegalese sole (*Solea senegalensis*) for accurate normalisation of immune gene expression during vaccination trials against NNV. Suitability of candidate genes was evaluated in juveniles by two statistical algorithms (geNorm and NormFinder), along with an integrated reference gene stability assessment tool for qPCR (RefFinder). This assessment was performed in three tissues selected for their roles in NNV replication (brain) and immune response (kidney and gut) in experimentally NNV-infected sole and controls. Furthermore, the suitability of the selected genes was evaluated in bath-vaccinated sole.

## 2. Methods

### 2.1. Fish

Senegalese sole juveniles with average body weights of 5 ± 0.1 g (used for the screening of the reference genes) and 1 ± 0.1 g (employed in the validation of the selected genes) were purchased from a local fish farm (Stolt Sea Farm S.L.). A total of 54 individuals (27 of each size) were housed at the aquarium facilities of the University of Santiago de Compostela (Spain) and randomly distributed into four 200 L opaque tanks (*n* = 12) containing seawater (salinity 33 g/L) at 18 °C. The fish were maintained under a 12:12 h light/dark photoperiod and were daily fed *ad libitum* with dry commercial pellets. All efforts were made to minimise animal suffering following the European Directive (2010/63/UE). Experimental protocols were approved (21 July 2020) by the Bioethics and Experimental Animal Welfare Committees of the University of Santiago de Compostela and Xunta de Galicia (Permit Id. 15.010/2020/004). Prior to the experiments, 3 fish from each batch were euthanised with tricaine methanesulfonate (MS-222; Sigma-Aldrich, St. Louis, MO, USA) overdose to confirm the absence of viral and bacterial pathogens, according to standard guidelines for aquatic animal health and diagnostic practices [16].

### 2.2. Experimental Infection and Sampling Procedure

Juveniles (5 g ± 0.1 g average body weight) were intramuscularly injected with 100 μL of a NNV strain (SpSsIAUSC160.03; [17]) at 5 × 10^5^ TCID_50_/mL (based on previous infections made by the group), or PBS (control group). Three individuals from each group were randomly sampled at 1, 3, 5, and 7 days post-challenge. Immediately upon sampling, three different tissues were collected: the brain, as the primary target of NNV [1]; the gut, commonly examined in oral vaccination studies [18]; and the kidney, the main immunogenic organ in fish [19]. Samples of each tissue (20 mg) were aseptically collected, processed individually by adding lysis buffer (Macherey-Nagel, Duren, Germany), homogenised mechanically and stored at −80 °C for further analysis.

### 2.3. Identification of Reference Genes in Senegalese Sole

Eight reference genes were selected based on the study of Infante et al. [20]: *β-actin* (paralogous genes *actb1* and *actb2*), *elongation factor 1 α* (*eef1a*), *glyceraldehyde-3P-dehydrogenase* (*gapdh2*), *hypoxanthine phosphoribosyltransferase* (*hprt1*), *ribosomal protein S4* (*rps4*), *ubiquitin* (*ubq*), and *18S ribosomal RNA* (*18S*). Accession numbers and additional details of the genes are summarised in Table 1.

### 2.4. RNA Isolation and Gene Expression Analysis

Total RNA was extracted from each tissue using the NucleoSpin^®^ Kit (Macherey-Nagel) following the manufacturer’s instructions. RNA was quantified in a Nanodrop ND-100 spectrophotometer (Nanodrop Technologies, Thermo Scientific, Waltham, MA, USA), and purity was given by the ratio 260/280 and 260/230. cDNA synthesis protocol started with RNA (~1 µg) incubation with random primers (200 nM) for 5 min at 95 °C. Then, reverse transcription mixture containing 200 U SuperScript™ IV Reverse Transcriptase (ThermoFisher, Thermo Scientific, Waltham, MA, USA), 0.5 mM dNTPs and 0.05 M DTT in 1× first strand buffer was added, and the final mixture (20 µL) was incubated at 25 °C for 10 min, followed by 50 min at 50 °C. The reaction was stopped by heating at 85 °C for 5 min.

The obtained cDNA was subjected to qPCR in a CFX-96 Real Time PCR Detection System (Bio-Rad, Hercules, CA, USA). Reaction mixture contained 10 μL of BlasTaq™ 2× qPCR MasterMix (abm) with 0.2 μM of the specific primers (Table 1) and 2 μL of cDNA template in 96-well plates using a final volume of 20 μL. Amplification was performed through a 3 min incubation at 95 °C (activation/denaturation step), followed by 40 cycles of 95 °C for 15 s and 60 °C for 30 s. Negative controls (containing nuclease-free water) were always included in the reactions. Each assay was performed in duplicate.

### 2.5. Statistics and Data Analysis

Three biological and two technical replicates were performed for each condition. Additionally, 9 samples were run in each reaction, representing inter-run calibrators. All graphs were generated in GraphPad Prism 8.0 (GraphPad Software Inc., La Jolla, CA, USA), except for the RankAggreg graphs, which were built in the R statistical programming environment (version 4.4.0). Data from both NNV-challenged and naïve fish were pooled and analysed together and are represented as mean ± SD (standard deviation). Comparison of means was carried out with the *t*-test and Mann–Whitney test using the statistical wizard of qBase plus. *p* values < 0.05 were considered statistically significant.

### 2.6. Expression Profile of the Reference Genes

Expression profile of the reference genes was evaluated by cycle threshold (Ct) values and melting curves. Ct represents the cycle number in which the generated fluorescence signals reach a detectable level. Hence, lower Ct values indicate higher expression levels, and higher Ct values are equivalent to low gene expression. Melting curves represent the denaturation of the dsDNA generated during the amplification and confirm that the reaction specifically amplifies the DNA target. A single peak indicates a single amplification product of the expected size.

### 2.7. Stability Analysis of the Candidate Reference Genes

Stability of the 8 candidate reference genes was analysed using three complementary tools. First, we applied the two main independent statistical algorithms: the geNorm module in qbaseplus version 2.5 [8], currently available through the Clarida platform (https://clarida.bio/tools/genorm, accessed on 7 August 2026) [22], and NormFinder in Microsoft Excel (https://www.moma.dk/software/normfinder; accessed on 7 August 2026 [2]). Subsequently, RefFinder (https://github.com/fulxie/RefFinder; accessed on 7 August 2026 [23]) was used to independently implement geNorm, NormFinder, BestKeeper, and ΔCt method, integrate the outputs of these algorithms, and generate a consensus ranking. For clarity:

- geNorm computes expression stability values for the panel of candidate reference genes [8] with respect to the M-value, which represents the average pairwise variation (V) of a given gene relative to all the panel. Generally, an M-value below 1.5 is considered acceptable for normalisation [4]. Although this algorithm assumes that none of the genes analysed are co-regulated [20], such co-regulation should not be ruled out. In our analysis, geNorm was applied using gene-specific PCR efficiencies to obtain more accurate stability estimates.
- NormFinder, a Visual Base Application for Microsoft Excel, estimates the stability on the expression of the candidate genes and provides a rank from the most stable gene to the least. This model-based approach takes into consideration both intergroup and intragroup variation and shows less sensitivity to co-regulation [2]. As with geNorm, NormFinder was run using gene-specific PCR efficiencies to improve the precision of stability calculations.
- RefFinder is an online tool that integrates the major four computational programs: geNorm, NormFinder, BestKeeper, and ΔCt [23], and has been previously applied to the selection of reference genes in fish [24]. BestKeeper tool compares expression levels of different candidate reference and target genes and determines the optimal genes by performing pairwise correlation analysis of the Ct values and calculating the geometric means of the most suitable ones [15]. ΔCt compares the relative expression of a pair of genes under each condition, and the most stable gene is ranked by the lowest arithmetic mean of the SD calculated across the samples [7]. Each of the four programs assigned an appropriate weight to each gene, and then RefFinder computed the geometric means to rank the most stable genes of the panel. The lower the geometric mean, the more stable and suitable the gene [23].

### 2.8. Integration of Ranking Lists by RankAggreg

None of the previously described algorithms is universally accepted as the single best option to evaluate and rank reference genes. To simplify interpretation by combining all algorithm outputs into a single, the individual rankings generated by geNorm and NormFinder, together with the consensus ranking provided by RefFinder were integrated by RankAggreg (version 0.6.6) in the R statistical programming environment. The cross-entropy Monte Carlo algorithm was used to calculate the weighted Kendal distance to identify the most stable genes in the three tissues analysed (brain, gut and kidney) in both control and treated fish. RankAggreg study is valuable when different algorithms produce partially divergent results, since formal optimisation methods, such as cross-entropy or genetic algorithms, generate more robust and unbiased final rankings [25].

### 2.9. Determination of the Number of Reference Genes Required for Accurate Normalisation

geNorm module selects the optimal number of genes for accurate normalisation via a stepwise comparison that excludes the least stable genes (highest M-values). A pairwise variation below 0.15 indicates that additional reference genes are not required. Once the most stable reference genes and the optimal number of genes are selected, normalisation factors are obtained based on the geometric mean of the expression levels of the best reference genes [8].

### 2.10. Validation of Reference Gene Stability

To preliminarily test these reference genes for vaccination studies, we quantified the expression of immune-related genes following immunisation. Fish with an average weight of 1 g were vaccinated by immersion with a recombinant NNV protein (10 µg/mL; [26]) or PBS (mock-vaccinated group) for 10 min. A 20 mg sample of anterior kidney—considered the main immunological organ in fish—was selected for analysis of three cytokine-coding gene expression. At each sampling point (1, 3, 5, and 7 days post-vaccination), three fish were randomly collected and processed individually. Relative expressions of the interleukine-1 beta (*il1β*), interleukine-6 (*il6*), and tumour necrosis factor α (*tnfα*) genes, which encode for key pro-inflammatory cytokines of the innate immune system [27] were quantified by geNorm, with the PBS group as the control group using the primers in Table 1. Data were analysed using the two most stable and least stable genes in anterior kidney.

## 3. Results

### 3.1. Detection of NNV

**Figure 1:**
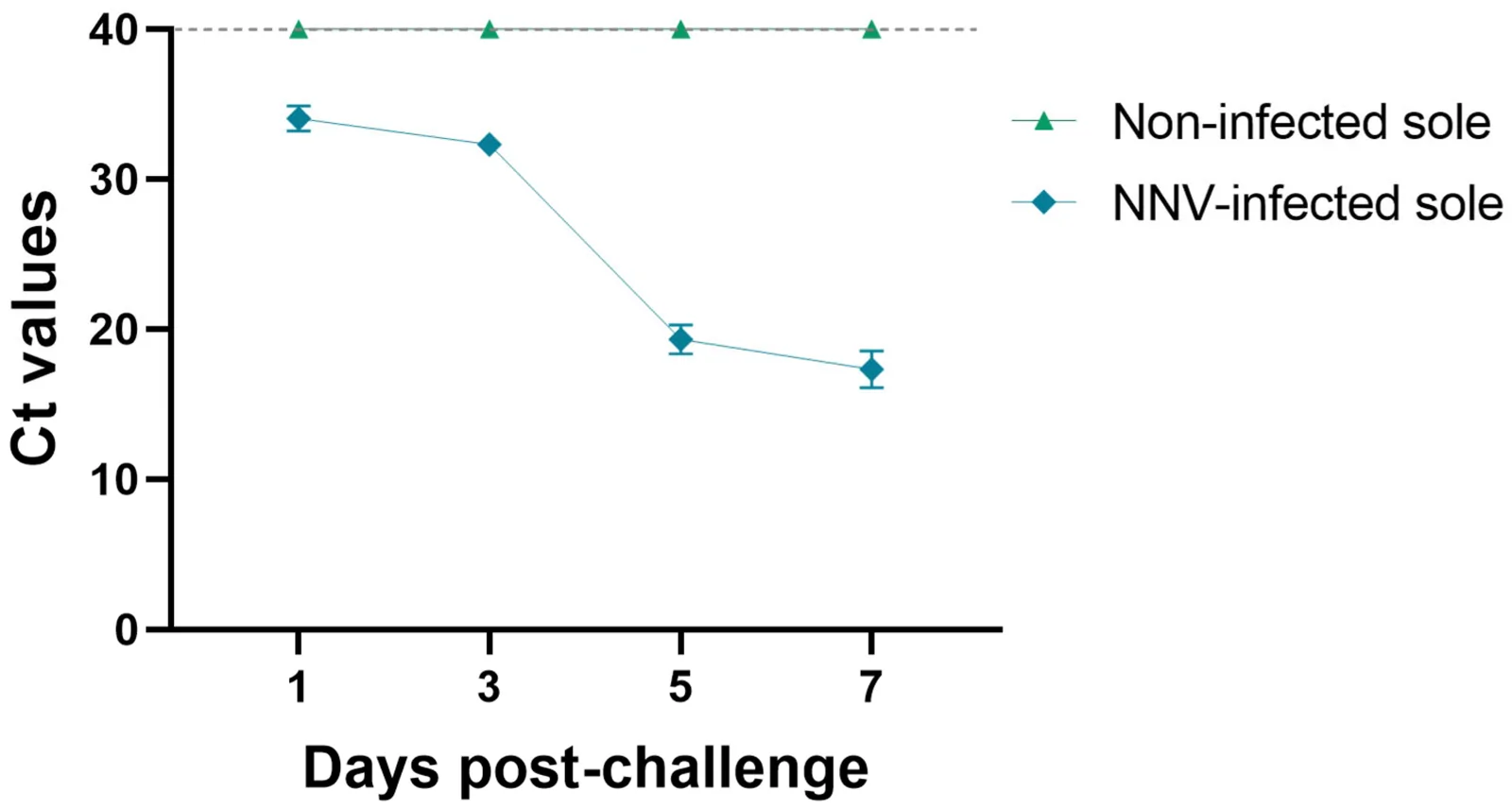
Detection of NNV in *Solea senegalensis* brain samples by RT-qPCR from NNV-infected and non-infected sole juveniles. Data are represented as average Ct values ± SD (n = 3). Samples that tested negative were assigned the maximum number of qPCR cycles (Ct = 40). Ct values inversely relate to the abundance of the NNV genome.

In order to obtain an accurate panel of stable genes whose expression remains stable regardless of viral infection in the fish, an experimental challenge was carried out. Viral load was assessed before determining the suitability of the eight candidate reference genes for normalisation. NNV was detected in the three biological replicates from the infected group in all time points analysed (Figure 1). At 24 h post-challenge, Ct values were on average 34.05 ± 0.82 and progressively decayed to 17.32 ± 1.22 on day 7 post-challenge, demonstrating an effective NNV replication. The NNV genome was not detected in any of the mock-infected fish.

### 3.2. Expression Profile of the Reference Genes: Ct and Melting Curves

Ct values of the tested reference genes from both control and NNV-infected fish ranged from 9.4 (*18S*) to 27.96 (*hprt1*) in the brain (Figure S1a), 8.86 (*18S*) and 31.21 (*hprt1*) in the gut (Figure S1b), and 9.06 (*18S*) and 31.98 (*hprt1*) in the kidney (Figure S1c). The lowest intragenic SD was observed in the kidney and brain, while the most variable expression was recorded in the gut (*eef1a*: 1.65; *hprt1*: 1.66). Overall, analysis of the expression levels of the eight genes in the three tissues indicated that average values in each gene ranged from 10.50 ± 0.69 (*18S*) to 28.43 ± 1.91 (*hprt1*) (Figure S1d). Detailed Ct values obtained for each gene in the brain, kidney and gut are plotted in the Supplementary Materials (Figures S2–S4, respectively).

The melting curves of amplified genes show a single amplification product of the expected size (Figure S5). No amplification nor melting curves were detected in non-template controls.

### 3.3. Evaluation of Gene Expression Stability by geNorm

**Figure 2:**
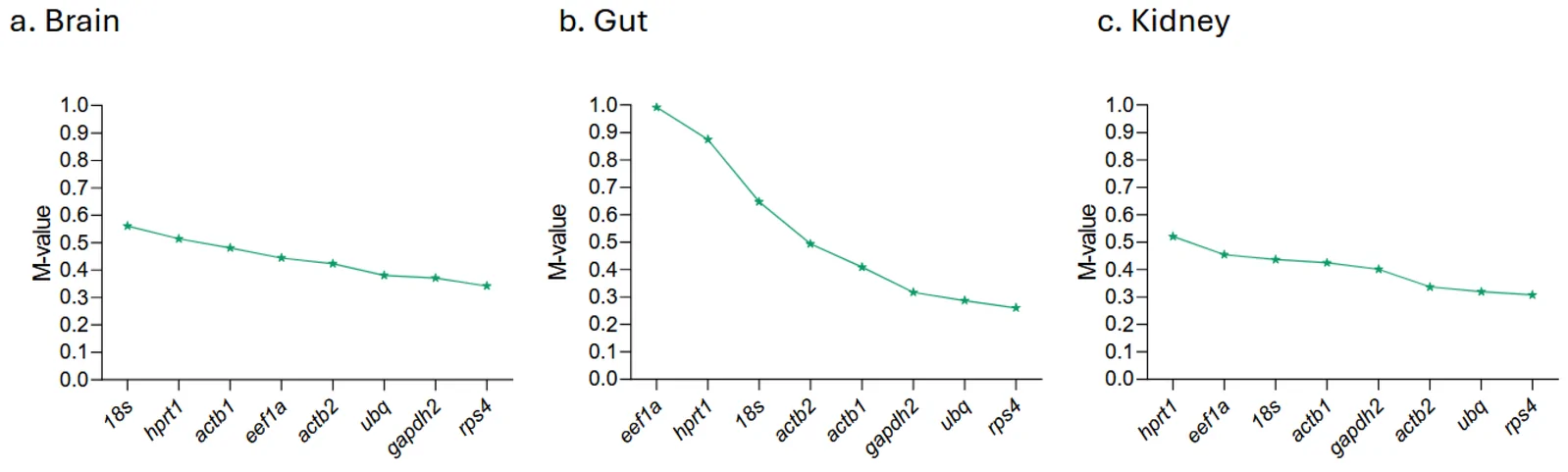
Average expression stability expressed as M-value of the 8 candidate HKGs according to geNorm, represented from the least stable to the most.

Expression stability of the candidate reference genes in geNorm was expressed as M-values, with the most stable genes showing the lowest values. In both control and NNV-infected fish, the most stable gene in all tissues was *rps4*, with M-values of 0.342 in the brain and 0.260 and 0.308 in the gut and kidney, respectively (Figure 2). In the gut and kidney, *rps4* was followed by *ubq* (0.287 and 0.319; respectively). Meanwhile, in the brain, the second most stable gene was *gapdh2* (0.371), although its stability was very similar to that of *ubq* (M = 0.381), which ranked third. All tested organs were analysed together, suggesting the following ranking (from the most stable to the least): *rps4* > *ubq* > *actb2* > *18S* > *gapdh2* > *actb1* > *eef1a* > *hprt1*. The M-value for the best combination of the two most stable genes (*rps4* and *ubq*) is 0.284 (CV = 0.098; coefficient of variation).

### 3.4. Evaluation of Gene Expression Stability by NormFinder

Expression stability of the candidate reference genes in NormFinder was expressed as M-value and standard error (SE), with the most stable genes showing the lowest M-values. In brain tissue, the ranking obtained with NormFinder was identical to that provided by geNorm, with *rps4* and *gapdh2* as the two most stable genes (M = 0.213 and 0.271, respectively; Table 2), followed by *ubq* (0.281). In the kidney, *rps4* also ranked first followed by *actb2* (M = 0.192 and 0.298, respectively), while *ubq* appeared further down the list, with an M-value of 0.381. The results in the gut differed from the previous tissues: the top two genes were *actb2* and *actb1* (M = 0.141 and 0.391), whereas *rps4* and *ubq* followed with very similar M-values (0.534 and 0.545).

### 3.5. Evaluation of Gene Expression Stability by BestKeeper in RefFinder

In Bestkeeper, stability of reference genes is ranked by the SD, the coefficient of variation (CV) and the correlation coefficient (r). The most stable genes are those with the smallest SD and CV, and greater r. According to Pfaffl et al. [15], a suitable gene is represented by SD values below 1.0. Our results show that the average SD value of all candidates in the three tissues was below 0.69, except for *eef1a* and *hprt1* in the gut, which showed an average SD of 1.33 (Table 3). According to this algorithm, the most stable genes in the brain are *gapdh2* and *18S* (SD = 0.39 and 0.47, respectively). In the gut, *rps4* and *18S* ranked highest (SD = 0.44) In the kidney, the two optimal reference genes are *eef1a* and *rps4* (SD = 0.24 and 0.44, respectively).

### 3.6. Evaluation of Gene Expression Stability by ΔCt in RefFinder

Stability of reference genes by ΔCt was ranked by the minimum SD of the Ct means across the samples. The lowest values were detected in the kidney samples, in which the SD for the two most stable genes was 0.57 (Table 4). In the brain, the top two genes in the rank were *rps4* and *gapdh2*, showing SDs of 0.62 and 0.63, respectively, while in the gut, paralogous genes *actb1* and *actb2* showed the lowest SD values (0.93 and 0.95, respectively).

### 3.7. Verification of Stability Ranking by RefFinder

RefFinder provides a comprehensive ranking indicating that the *rps4* gene is the most stable in the brain and kidney (geometric mean 1.32 and 1.68, respectively), and *actb2* in the gut (geometric mean of 1.97) (Table 5).

### 3.8. Integration of Ranking Lists by RankAggreg

**Figure 3:**
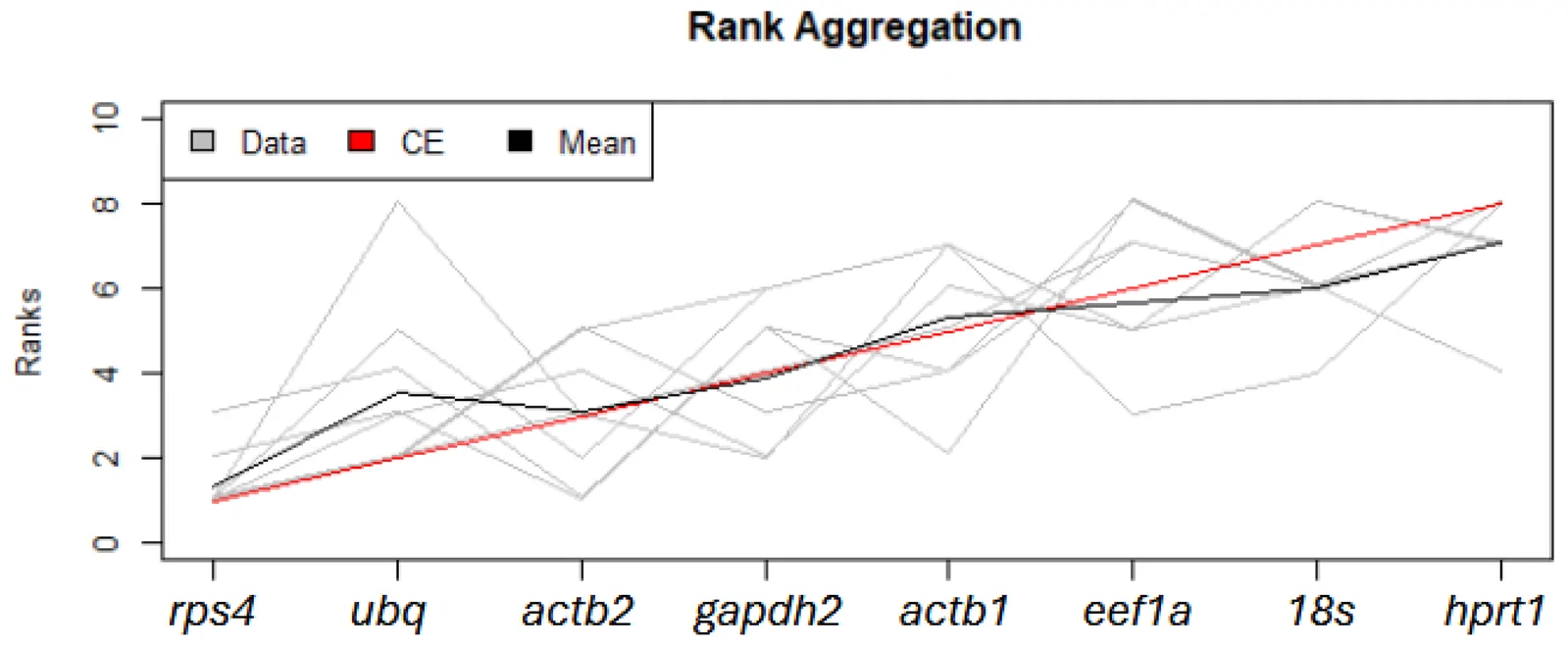
Rank aggregation analysis of the 8 candidate reference genes, based on the rank positions stablished by geNorm, NormFinder and RefFinder, in the 3 tissues analysed (brain, gut and kidney) shown in grey lines. Black line: mean rank position of each gene. Red line: model computed by the Monte Carlo algorithm. Some grey lines overlap because ranking profiles are identical or highly similar.

Considering the results of the stability analysis provided by geNorm, NormFinder and RefFinder, we performed a new ranking by RankAggreg that compiled all data from the three tissues. As a result, the following rank was obtained (from the most stable to the least): *rps4* > *ubq* > *actb2* > *gapdh2* > *actb1* > *eef1a* > *18S* > *hprt1* (Figure 3). These results are supported by the data obtained for each tissue and analysed individually (Figure S6).

### 3.9. Determination of the Number of Reference Genes Required for Accurate Data Normalisation by geNorm

**Figure 4:**
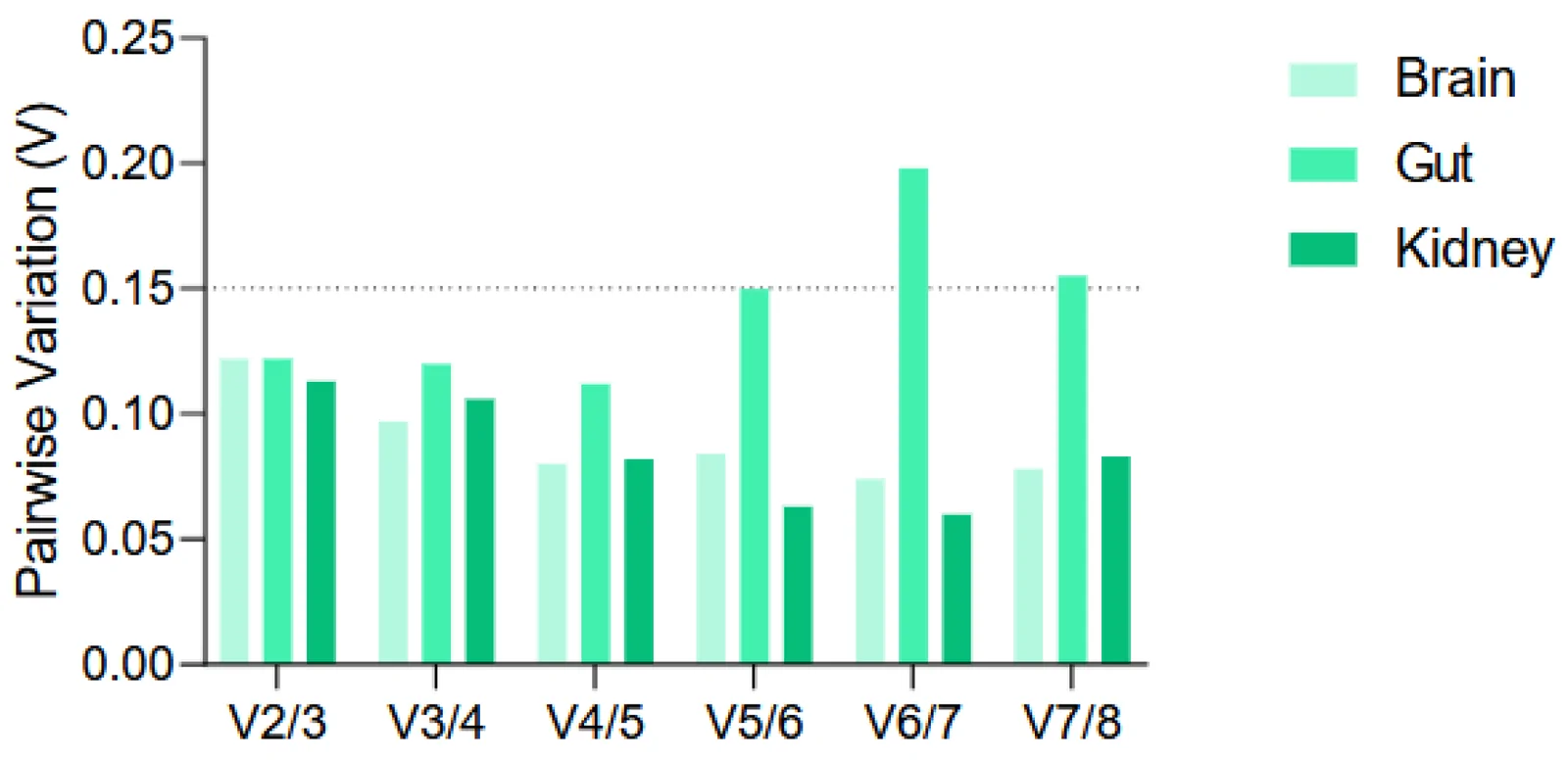
Pairwise variation (V) results given by geNorm in the brain, gut and kidney, including control and treated fish. The V-values were calculated using the normalisation factors NFn and NFn + 1. Line at Y = 0.15 represents the cut-off value.

The optimal number of reference genes used for data normalisation was calculated by geNorm using the pairwise variation (V). The cut-off value was set at 0.15. Our results for each tissue analysed separately indicate that two genes are enough to obtain accurate normalisation (Figure 4), since the value was below the cut-off and the inclusion of a third gene (V2/3) did not result in a significantly reduction. The V2/3 value of the brain and gut samples was 0.122, and the inclusion of a third reference gene slightly reduced the pairwise variation (V3/4 = 0.097 and 0.120, respectively). In kidney, the V2/3 value was 0.113, and the inclusion of a third reference gene yielded a V3/4 value of 0.106.

Therefore, geometric averaging of the two most stable genes in the three tissues in both control and treated fish is appropriate for accurate normalisation. These results are supported by the data obtained for control and treated fish and analysed separately (Figure S7).

### 3.10. Evaluation of the Genes Selected for Normalisation During Vaccination Assays in Early Juvenile Sole

**Figure 5:**
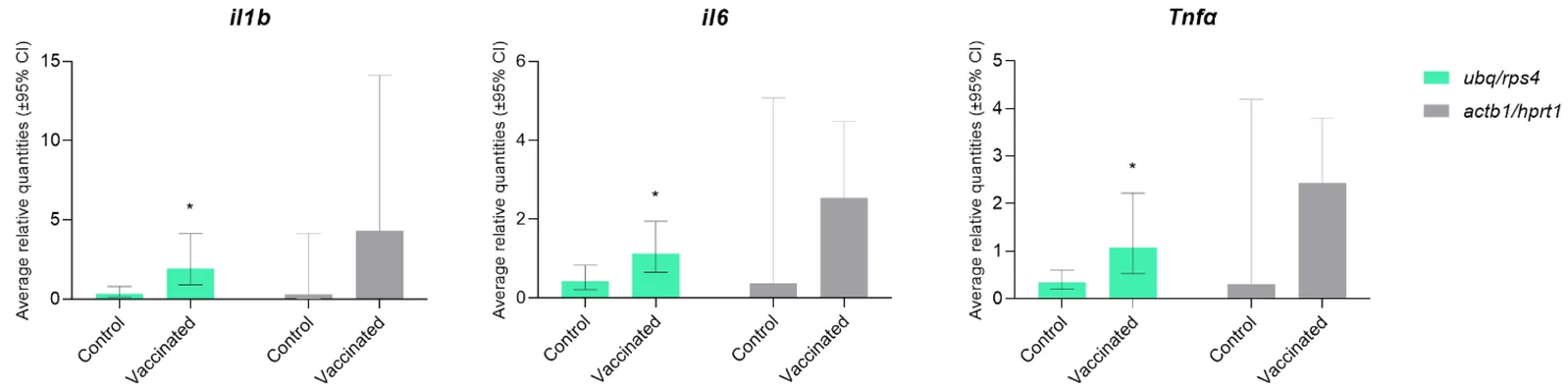
Relative expression levels of *il1b*, *il6*, and *tnfα* at 7 days post-vaccination in vaccinated and control fish. Data were normalised using either the two highest-ranked reference genes (green) or the two lowest-ranked reference genes (grey). Results are presented as the group mean, with error bars indicating the lower and upper bounds of the 95% confidence interval of the mean. Asterisk indicates significant differences between vaccinated and control groups (*p* < 0.05).

Considering the gene ranking in kidney, we evaluated the vaccinated fish using both the highest-ranked and the lowest-ranked reference genes. Expression of *il1β*, *il6*, and *tnfα* (Figure 5) was significantly upregulated in vaccinated fish with respect to the mock-vaccinated group (*p* value < 0.05) when *rps4* and *ubq* were used as reference genes (M = 0.335; CV = 0.117). In contrast, normalisation with *actb1* and *hprt1* genes yielded an M-value close to the accepted threshold (1.337 vs. 1.5) and a markedly higher CV (48.7% vs. 11.7%), and consequently, no significant differences were detected. The high variability among samples prevented the identification of statistically significant differences between groups.

## 4. Discussion

Quantifying the expression of genes related to the immune response following fish vaccination is one of the main steps for evaluating vaccine efficacy. Reliable RT-qPCR data require the use of internal control genes with stable expression, which must be validated for each fish species and experimental condition. This validation should include multiple tissues and physiological conditions to ensure the selection of the most appropriate genes [3]. In this study, we evaluated the suitability of eight commonly used reference genes in three tissues (brain, kidney and gut) of juvenile Senegalese sole following infection with NNV and compared the results with those from non-infected controls. This approach is particularly relevant because the stability of reference genes could be affected during NNV infection due to the up-regulation of pro-apoptotic genes in necrotic tissues, as observed in Atlantic halibut [28]. We carried out the analyses using the two most widely applied algorithms, geNorm and NormFinder, independently, as well as the web-based tool RefFinder, which integrates major computational programs (geNorm, NormFinder, BestKeeper, and the comparative ΔCt method) to overcome the constraints of each individual method while benefiting from their combined strengths. Notably, geNorm and NormFinder transform Ct values into relative quantities, whereas RefFinder relies exclusively on raw Ct values and assumes 100% PCR efficiency for all genes [23]. As demonstrated by De Spiegelaere et al. [29], this simplification can lead to gene stability rankings that differ from those generated by the original implementations of geNorm and NormFinder implementations, particularly when gene-specific efficiencies are considered, as in our analysis. To obtain a more robust and comprehensive evaluation, all results were subsequently integrated using RankAggreg, which improves ranking accuracy and mitigates algorithm specific biases [25]. It should be noted that geNorm and NormFinder are included twice in this procedure, which may bias the aggregated ranking toward the algorithms that are represented more than once and reduce the independence of the inputs. However, this duplication is unlikely to affect the identification of the most and least stable genes and would mainly introduce variation among those with intermediate stability. According to our data, RankAggreg identified *rps4*, *ubq* and *actb2* as the three most stable genes in the three analysed tissues. In contrast, *eef1a*, *18S* and *hprt1* are the least stable, while *gapdh2* and *actb1* showed intermediate stability.

The ribosomal *rps4* gene was identified as the most stable among all tissues and conditions, which could be due to its fundamental role in ribosomal functions. Suitability of this gene for normalisation has already been demonstrated in different larval stages of Senegalese sole and halibut (*Hippoglossus hippoglossus*) [20], and in turbot (*Scopthalmus maximus*) gonad [30]. Ribosomal genes are commonly used for normalisation of genes that display high expression values [7]. The use of *18S* as a reference gene has shown inconsistent reliability. This gene has been extensively used to assess immune-related gene expression [31,32], and several studies have reported its high stability in species such as zebrafish (*Danio rerio*) and Japanese flounder (*Paralichthys olivaceus*) [33,34]. However, studies in bivalves have demonstrated that *18S* exhibits the lowest stability among several candidate reference genes, underscoring that its reliability is context-dependent and should not be assumed without prior validation [35]. Likewise, our results indicate that *18S* is among the least suitable candidates for data normalisation. Under our experimental conditions *18S* showed a Ct value of 10.50 ± 0.69, which is considerably lower than the range generally recommended for reliable qPCR normalisation. Moderately abundant templates with Ct values above 15 are preferred for robust quantification, as very low Ct values often indicate template saturation or inhibitory effects [36].

*ubq*, which encodes ubiquitin, ranked the second in overall stability, in line with the findings of Infante et al. [20] and Robledo et al. [30] in Senegalese sole, halibut and turbot. Its expression remained stable in kidney and gut tissues; however, rankings in brain tissue varied across algorithms, highlighting the importance of integrating results from multiple approaches. This gene has been used as a reference gene in the assessment of the immune response against viral infections in different fish species [32,37]. Although *ubq* is a highly conserved gene, its expression has been reported to be modulated in response to different stressors in yeast, birds and mammals [38]. In our study, the variation in the brain is mainly driven by the fact that, amongst the set of genes analysed, this gene shows the highest SD and CV in BestKeeper (SD = 0.65; CV = 3.11%).

The paralogous gene *actb2* of *β*-*actin*, coding for cytoskeleton proteins, was also in the top three stable genes, although different stability values were provided by each of the algorithms tested. *β*-*actin* genes are frequently used as internal controls, but its unsuitability as reference genes has already been evidenced [39]. Our data indicate similar stability values of the two *β*-*actin* paralogous genes and *gapdh2*. *gapdh2* is involved in the glycolytic pathway and considered suitable as a reference gene in Senegalese sole larvae during different developmental stages and in Japanese flounder [20,40]. However, studies in cod and halibut indicated that this gene is unsuitable as a reference for data normalisation because it is related to several cellular functions, and therefore, the expression is dependent on the cell cycle and varies among different developmental stages, tissues, or physiological conditions [12,28,39,41,42,43]. Based on our data, its suitability appears to be restricted to brain tissue.

Conversely, genes related to protein metabolism (*eef1a*) and purine regulation (*hprt1*) were among the least stable. Although *eef1a* has been identified as one of the best candidate reference genes for normalisation in halibut larvae and juveniles and in Senegalese sole larvae [20,28], its expression can be co-regulated by ribosomal proteins, and therefore, its suitability as a reference gene is questionable [44]. Although it showed good performance in kidney (SD = 0.24; CV = 1.11), it was not consistent across the set of tissues. Finally, *hprt1* showed a very low expression (Ct value around 30) making it not appropriate as an internal reference, since expression levels of the target and the internal gene should be similar for accurate normalisation [7]. *hprt1* is considered the least stable gene in the three tissues analysed, with standard deviation values exceeding 1.

Accurate normalisation of gene expression requires determining the optimal number of reference genes. According to geNorm, two genes are sufficient for reliable normalisation, as the pairwise variation (V2/3) of the two most stable genes is below the recommended cut-off value (0.15), and the addition of further reference genes does not significantly improve the reliability of the normalisation factor. These findings are consistent with previous studies suggesting that a minimal set of reference genes helps reduce technical noise while preserving biological significance [45]. Based on our results, the *rps4* and *ubq* genes are adequate for gene normalisation in brain, gut, and kidney tissues of juvenile Senegalese sole. Alternatively, *ubq* can be replaced by *gapdh2* in brain tissue. Immune-related transcriptomic responses of Senegalese sole have been studied previously, reporting high modulation of different genes after exposure to toxins and antigens in sole adults [46]. However, the analysis of immune-related gene modulation during early developmental stages is often constrained, primarily due to the limited amount of tissue available from small fish and the inherently low immune responsiveness at these stages [47].

To evaluate the suitability of *rps4* and *ubq* genes, we assessed gene expression of the pro-inflammatory cytokines *il1β*, *il6*, and *tnfα* in anterior kidney of vaccinated and control fish. When normalising gene expression with the stable reference genes *rps4* and *ubq*, subtle changes in the expression profile of the pro-inflammatory cytokines were successfully detected. In contrast, the combination of *hprt1* and *actb1* genes for normalisation showed unstable values, indicating that these genes are not suitable as references. Thus, this highlights the need for highly stable reference genes to ensure robust and meaningful relative quantification that can be applied in vaccine development studies.

Based on this study, the proposed reference genes are considered stable for juvenile sole weighing 1–5 g, which corresponds to the size range included in the present analysis. Because the major morphophysiological changes associated with metamorphosis have already been completed at this stage, it is plausible that these genes may also exhibit comparable expression stability in adult sole. However, this hypothesis was beyond the scope of the present study and would require further experimental validation.

## 5. Conclusions

This study evaluated the expression stability of eight commonly used reference genes in the brain, kidney, and gut of juvenile Senegalese sole, using the two most widely used algorithms: geNorm and NormFinder, and the integrative tool RefFinder. Based on the integrated results, *rps4* and *ubq* were identified as the most suitable reference genes in sole under normal physiological conditions and following NNV infection, whereas *gapdh2* can be used as an alternative to *ubq* for brain analyses. The observed variability in gene stability across the three tissues points to the importance of potential reference gene validation before performing vaccination trials because there is no gold standard for gene expression normalisation. Normalisation with the most stable genes enabled the detection of subtle changes in the expression of the target genes. Finally, integrating data from naïve fish and fish infected with NNV at various post-infection time points provides evidence that these reference genes are suitable for vaccination studies.

## Figures and Tables

**Table 1:** Candidate reference genes from *Solea senegalensis* and primers used for RT-qPCR. E: efficiency of the primers in RT-qPCR.

Gene	Gene Name	Primer Sequence	Amplicon Size (bp)	E	Accession No. Reference or Unigene ID
*actb1*	Actin, beta (Isoform 1)	F: 5′-CTCCATCGTCCACCGCAAGTGCTTC-3′	113	90%	AB360593 [20]
		R: 5′-TGTCCATTCGTGCAAGATCGGGAGA-3′			
*actb2*	Actin, beta (Isoform 2)	F: 5′-AATCGTGACCTCTGCTTCCCCCTGT-3′	92	96%	DQ485686 [20]
		R: 5′-TCTGGCACCCCATGTTACCCCATC-3′			
*eefa1I*	Elongation factor 1 alpha (Isoform 1)	F:5′-GATTGACCGTCGTTCTGGCAAGAAGC-3′	142	95%	AB326302 [20]
		R: 5′-GGCAAAGCGACCAAGGGGAGCAT-3′			
*gapdh2*	Glyceraldehyde-3-phosphate dehydrogenase	F: 5′-AGCCACCGTGTCGCCGACCT-3′	107	95%	AB291587 [20]
		R: 5′-AAAAGAGGAGATGGTGGGGGGTGGT-3′			
*hprt1*	Hypoxantine phosphoribosyl transferase	F: 5′-GGACATGGCGACATCCAGCTCCTGTGTT-3′	120	96%	AB360596 [20]
		R: 5′-TGGGGGATGTAGACCCTCTCCAGGTCAG-3′			
*rps4*	40S ribosomal protein S4	F: 5′-GTGAAGAGCTCCTTGTCGGCACCA-3′	83	94%	AB291557 [20]
		R: 5′-AGGGGGTCGGGGTAGCGGATG-3′			
*ubq*	Ubiquitin	F: 5′-AGCTGGCCCAGAAATATAACTGCGACA-3′	93	95%	AB291588 [20]
		R: 5′-ACTTCTTCTTGCGGCAGTTGACAGCAC-3′			
*18S*	*18S* ribosomal	F: 5′-GAATTGACGGAAGGGCACCACCAG-3′	148	97%	AM882675 [20]
		R: 5′-ACTAAGAACGGCCATGCACCACCAC-3′			
*NNV-RdRp*	NNV Viral polymerase	F: 5′-TCCAAAAGAAAGAAGCATAC-3′	140	96%	FJ803911 [21]
		R: 5′-TGGCATGTACCACGGAAC-3′			
*il1β*	Interleukin-1 beta	F: 5′-TGGAGGAATGTGACGAGGAG-3′	113	95%	solea_v4.1_UNIGENE66490
		R: 5′-GCACTTCACAGACTCGAACC-3′			
*il6*	Interleukin-6	F: 5′-GTGCGTGACATCTACAACCC-3′	127	97%	solea_v4.1_UNIGENE631474
		R: 5′-GCCGTCTTCTCCTTTCCCTT-3′			
*tnfα*	Tumour necrosis factor alpha	F: 5′-TGTGTACATGGGAGCTGTGT-3′	126	95%	XM_044052089
		R: 5′-CACAGAGCGAACACACCAAA-3′			

**Table 2:** Stability values (M), standard error (SE) and ranking order of the 8 candidate genes in *Solea senegalensis* analysed by NormFinder in the brain, gut, and kidney.

Ranking	Brain			Gut			Kidney		
	Gene	M	SE	Gene	M	SE	Gene	M	SE
1	*rps4*	0.213	0.037	*actb2*	0.141	0.093	*rps4*	0.192	0.035
2	*gapdh2*	0.271	0.039	*actb1*	0.391	0.067	*actb2*	0.298	0.040
3	*ubq*	0.286	0.040	*rps4*	0.534	0.074	*eef1a*	0.362	0.045
4	*actb2*	0.415	0.050	*ubq*	0.545	0.075	*18S*	0.371	0.045
5	*eef1a*	0.422	0.050	*gapdh2*	0.757	0.091	*ubq*	0.381	0.046
6	*actb1*	0.486	0.056	*18S*	0.888	0.102	*gapdh2*	0.410	0.048
7	*hprt1*	0.573	0.063	*hprt1*	1.203	0.131	*actb1*	0.439	0.051
8	*18S*	0.627	0.068	*eef1a*	1.250	0.135	*hprt1*	0.677	0.073

**Table 3:** Ranking order of the 8 candidate genes in *Solea senegalensis*, analysed by BestKeeper, in the brain, gut, and kidney.

**BRAIN**						
**Ranking**	**Gene**	**SD**	**CV**	**SD [Fold]**	**Coeff. of Corr. [r]**	***p*** **-Value**
1	*gapdh2*	0.39	2.23	1.31	0.714	0.001
2	*18S*	0.47	4.72	1.38	0.460	0.001
3	*rps4*	0.50	2.43	1.41	0.762	0.001
4	*actb1*	0.53	2.90	1.44	0.426	0.003
5	*hprt1*	0.55	2.10	1.46	0.638	0.001
6	*eef1a*	0.58	2.97	1.50	0.670	0.001
7	*actb2*	0.59	3.08	1.51	0.765	0.001
8	*ubq*	0.65	3.11	1.57	0.828	0.001
**GUT**						
**Ranking**	**Gene**	**SD**	**CV**	**SD [Fold]**	**Coeff. of Corr. [r]**	***p*** **-Value**
1	*rps4*	0.44	2.32	1.36	0.536	0.001
2	*18S*	0.44	4.39	1.36	0.384	0.007
3	*actb2*	0.45	2.25	1.36	0.792	0.001
4	*ubq*	0.58	3.03	1.50	0.612	0.001
5	*gapdh2*	0.60	3.08	1.52	0.376	0.008
6	*actb1*	0.65	3.68	1.56	0.703	0.001
7	*eef1a*	1.33	6.44	2.51	0.791	0.001
8	*hprt1*	1.33	4.78	2.52	0.807	0.001
**KIDNEY**						
**Ranking**	**Gene**	**SD**	**CV**	**SD [Fold]**	**Coeff. of Corr. [r]**	***p*** **-Value**
1	*eef1a*	0.24	1.11	1.18	0.652	0.001
2	*rps4*	0.44	2.27	1.35	0.797	0.001
3	*ubq*	0.50	2.60	1.41	0.879	0.001
4	*gapdh2*	0.57	3.11	1.48	0.749	0.001
5	*actb2*	0.58	3.33	1.49	0.779	0.001
6	*18S*	0.61	5.96	1.52	0.919	0.001
7	*hprt1*	0.61	2.04	1.53	0.541	0.001
8	*actb1*	0.69	3.77	1.62	0.818	0.001

**Table 4:** Ranking order of the 8 candidate genes in *Solea senegalensis*, analysed by ΔCt, in the brain, gut, and kidney, using RefFinder.

Ranking	Brain		Gut		Kidney	
	Gene	SD	Gene	SD	Gene	SD
1	*rps4*	0.62	*actb2*	0.93	*ubq*	0.57
2	*gapdh2*	0.63	*actb1*	0.95	*rps4*	0.57
3	*actb2*	0.70	*ubq*	0.97	*18S*	0.61
4	*eef1a*	0.71	*rps4*	0.98	*eef1a*	0.63
5	*hprt1*	0.72	*gapdh2*	1.06	*actb2*	0.63
6	*ubq*	0.73	*18S*	1.19	*gapdh2*	0.68
7	*actb1*	0.82	*eef1a*	1.40	*actb1*	0.73
8	*18S*	0.89	*hprt1*	1.47	*hprt1*	0.81

**Table 5:** Stability values and ranking order of the 8 candidate genes in *Solea senegalensis* analysed by RefFinder in the brain, gut, and kidney.

Ranking	Brain		Gut		Kidney	
	Gene	Geometric Mean	Gene	Geometric Mean	Gene	Geometric Mean
1	*rps4*	1.32	*actb2*	1.97	*rps4*	1.68
2	*gapdh2*	1.41	*rps4*	2.00	*ubq*	1.73
3	*actb2*	3.98	*ubq*	2.45	*eef1a*	2.11
4	*hprt1*	4.61	*actb1*	3.13	*18S*	4.05
5	*eef1a*	4.68	*gapdh2*	4.40	*actb2*	4.47
6	*18S*	5.66	*18S*	4.56	*gapdh2*	5.42
7	*actb1*	6.09	*eef1a*	7.00	*actb1*	7.24
8	*ubq*	6.16	*hprt1*	8.00	*hprt1*	7.74

## Data Availability

The original data presented in the study are openly available in Zenodo at 10.5281/zenodo.17367550.

## Supplementary Materials

The following supporting information can be downloaded at https://www.mdpi.com/article/10.3390/vetsci13080790/s1: Figure S1. Transcriptional levels in brain (a), gut (b) and kidney (c), and a combined analysis of the 3 tissues (d) of candidate reference genes expressed as Ct values. The combined analysis shown in (d) was generated by statistically averaging the Ct values obtained from the individual tissue samples after RT-qPCR. Ct values were plotted as means ± SD of two technical and three biological replicates at each time point. Figure S2. Expression levels of candidate reference genes in brain samples of control (*n* = 3) and treated (*n* = 3) fish for each time point, represented in a box-and-whisker plot. Boxes represent the 25th to 75th percentiles of Ct range, and whiskers represent the medians, showing the maximum and minimum values. Figure S3. Expression levels of candidate reference genes in kidney samples of control (*n* = 3) and treated (*n* = 3) fish for each time point, represented in a box-and-whisker plot. Boxes represent the 25th to 75th percentiles of Ct range, and whiskers represent the medians, showing the maximum and minimum values. Figure S4. Expression levels of candidate reference genes in gut samples of control (*n* = 3) and treated (*n* = 3) fish for each time point, represented in a box-and-whisker plot. Boxes represent the 25th to 75th percentiles of Ct range, and whiskers represent the medians, showing the maximum and minimum values. Figure S5. Melting curves of candidate reference genes. Figure S6. Integrated stability ranking by RankAggreg, using the data obtained from geNorm, NormFinder and RefFinder tools, for brain (a), gut (b) and kidney (c). Black line: Mean rank position of each gene. Red line: Consensus ranking (CE) generated by the Monte Carlo algorithm. Grey lines: Connect the ranking positions of the eight candidate reference genes across geNorm, NormFinder, and RefFinder for each tissue. Some grey lines overlap because ranking profiles are identical or highly similar. Figure S7. Pairwise variation (V) results given by geNorm in brain (a), gut (b) and kidney (c). The V-values were calculated using the normalisation factors NFn and NFn + 1. Line at Y = 0.15 represents the cut-off value.
