# Supplementary material for: Screening of Stable Reference Genes in Solea senegalensis Juveniles for Nervous Necrosis Virus (NNV) Vaccination Assays
Source: Vet Sci. 2026 Aug 8;13(8):790. doi: 10.3390/vetsci13080790 (PMC13517329; doi:10.3390/vetsci13080790)
Supplement: Supplementary file 1 [file vetsci-13-00790-s001.zip › vetsci-4424282-supplementary.pdf]

Figure S1

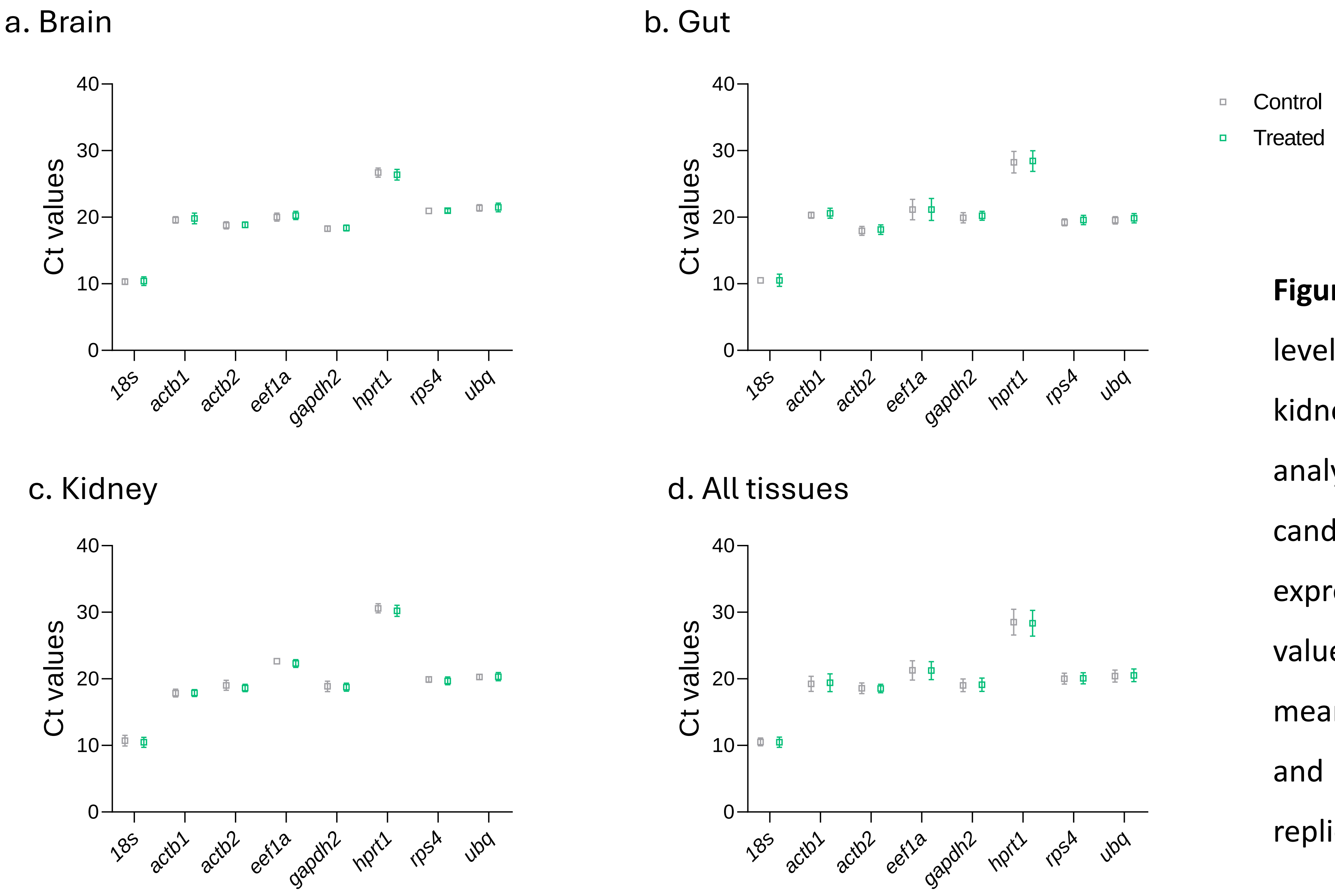

Figure S1. Transcriptional levels in brain (**a**), gut (**b**) and kidney (**c**), and a combined analysis of the 3 tissues (**d**) of candidate reference genes expressed as Ct values. Ct values were plotted as means ± SD of two technical and three biological replicates in each timepoint.

Figure S2

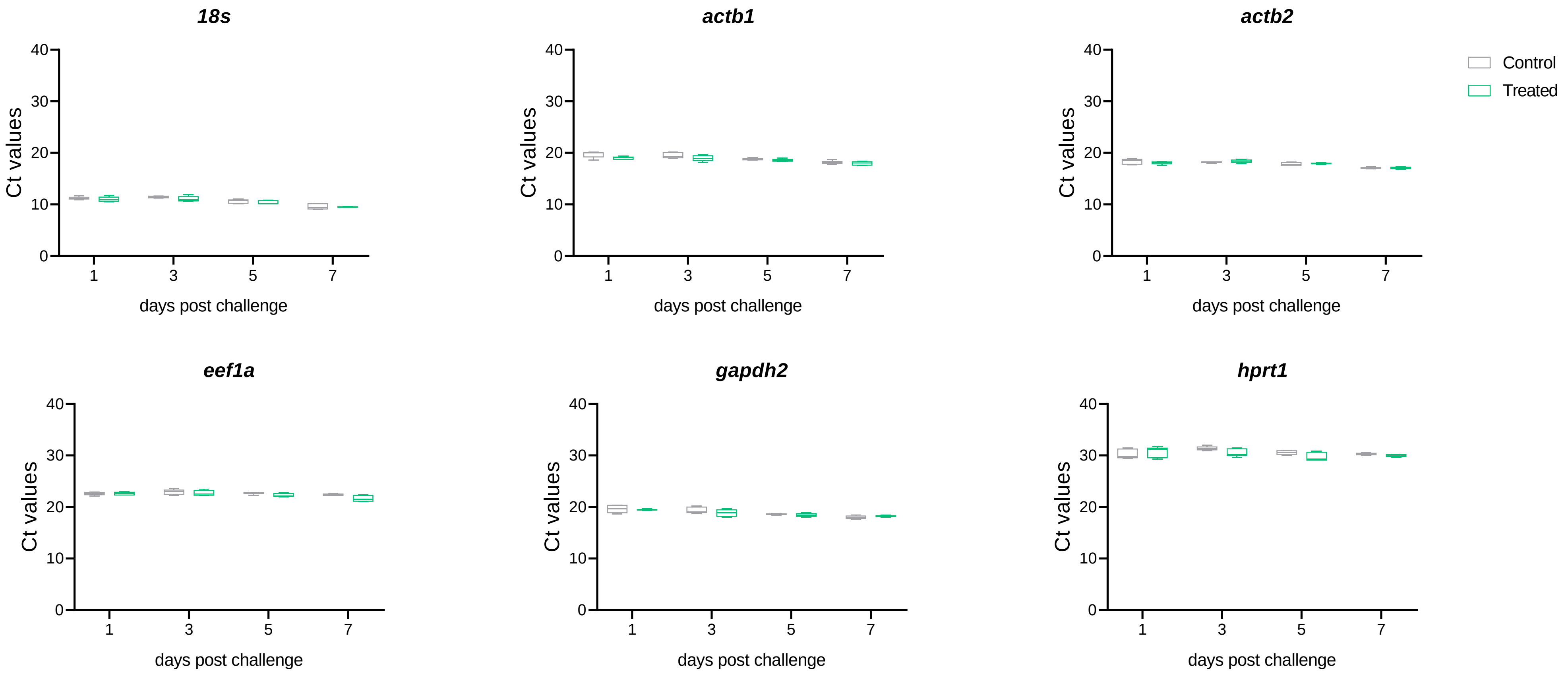

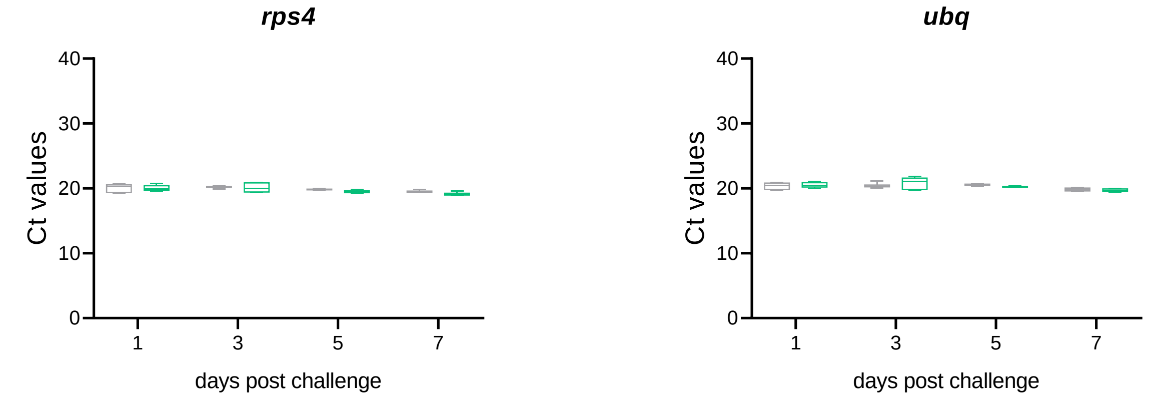

Figure S2. Expression levels of candidate reference genes in brain samples of control and treated and control fish represented in a box-and-whisker plot. Boxes represent the 25th to 75th percentiles of Ct range, and whiskers the medians showing the maximum and minimum values.

Figure S3

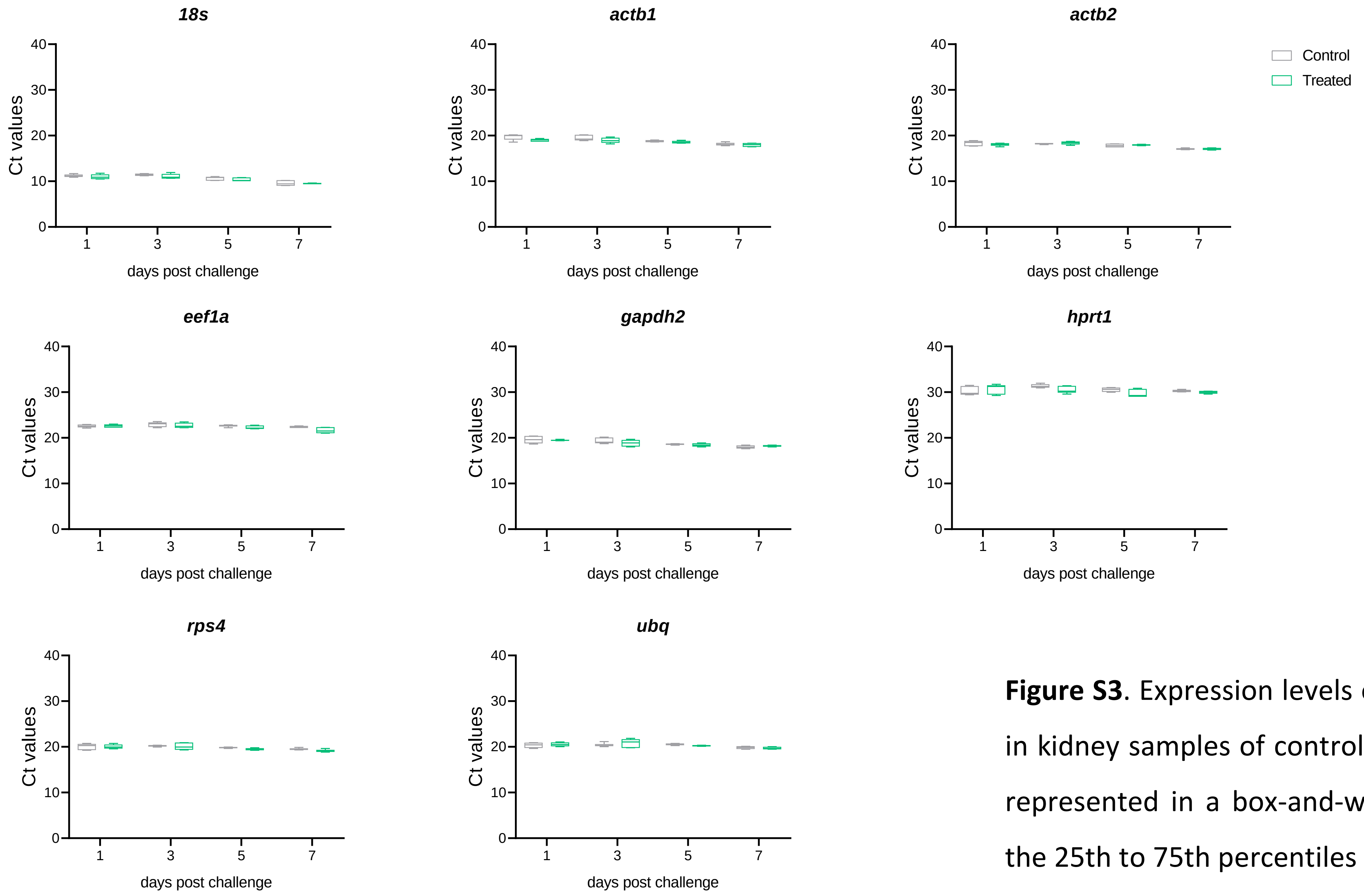

Figure S3. Expression levels of candidate reference genes in kidney samples of control and treated and control fish represented in a box-and-whisker plot. Boxes represent the 25th to 75th percentiles of Ct range, and whiskers the medians showing the maximum and minimum values.

Figure S4

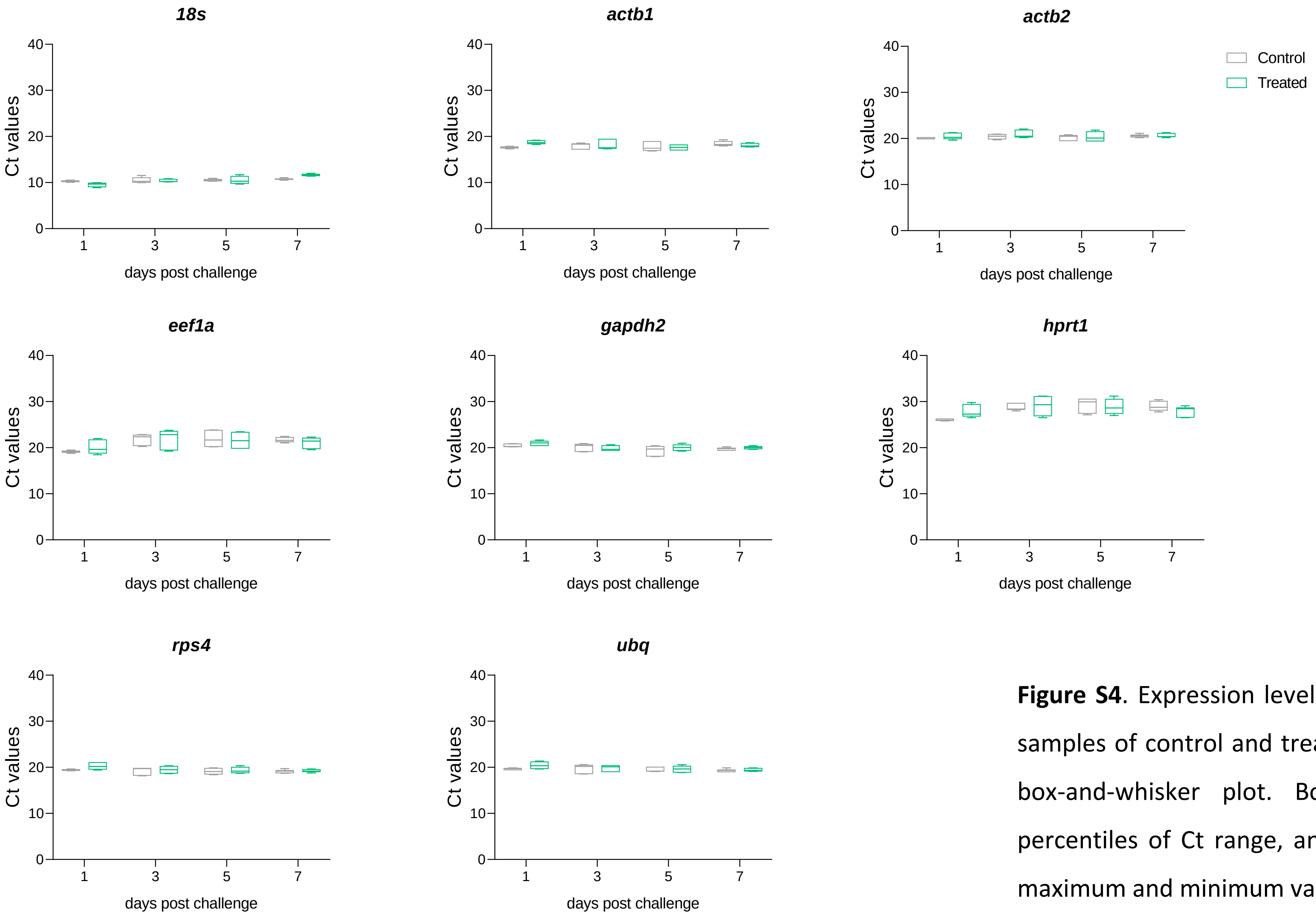

Figure S4. Expression levels of candidate reference genes in gut samples of control and treated and control fish represented in a box-and-whisker plot. Boxes represent the 25th to 75th percentiles of Ct range, and whiskers the medians showing the maximum and minimum values.

Figure S5

Figure S5. Melting curves of candidate reference genes.

a. *actb*1

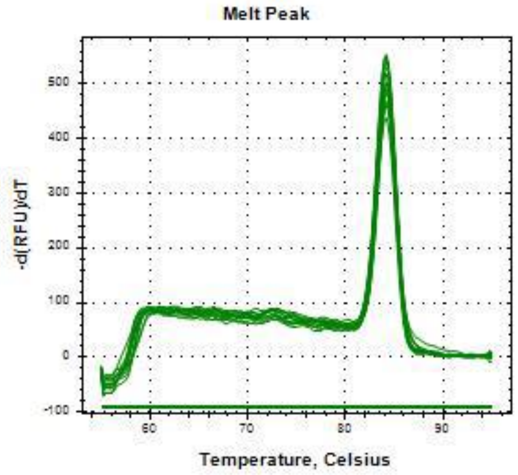

b. *actb*2

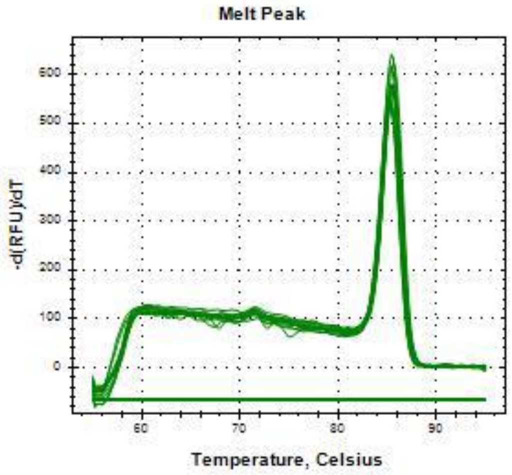

c. *eef*1*a*

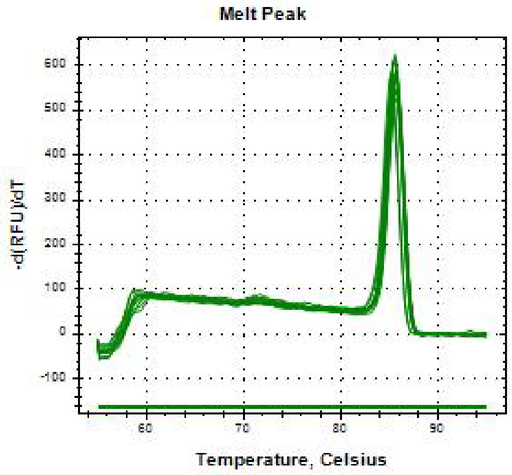

d.*gadph*

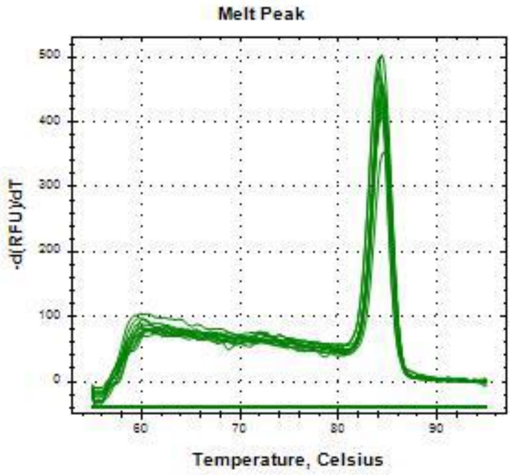

e. *hprt*1

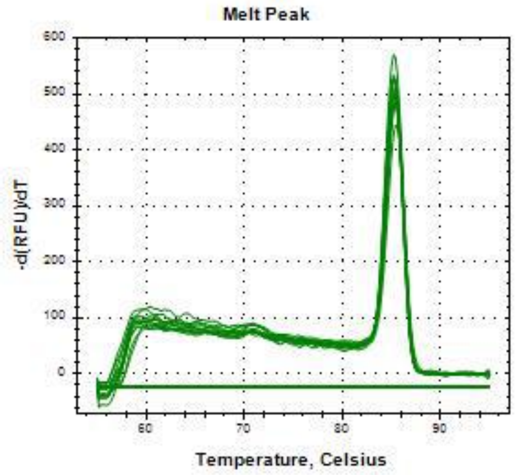

f. *rps*4

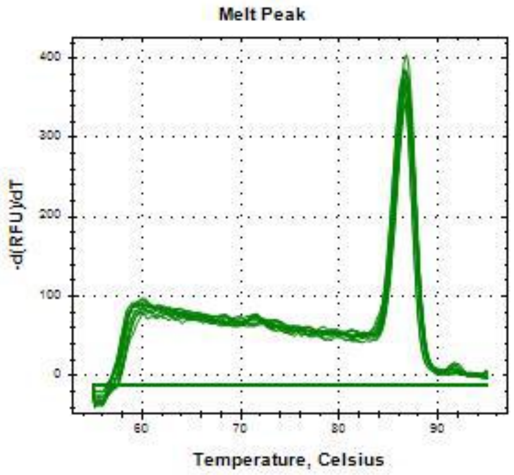

g. *ubq*

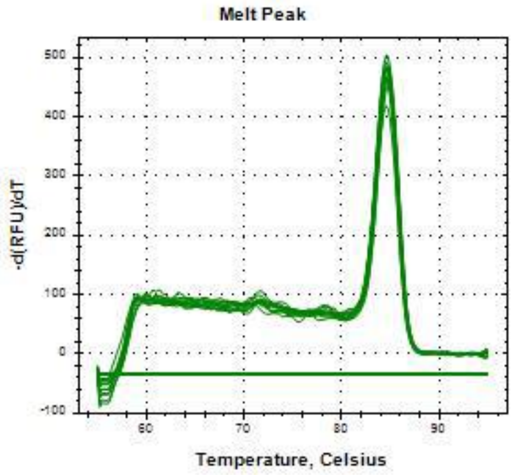

h.18*s*

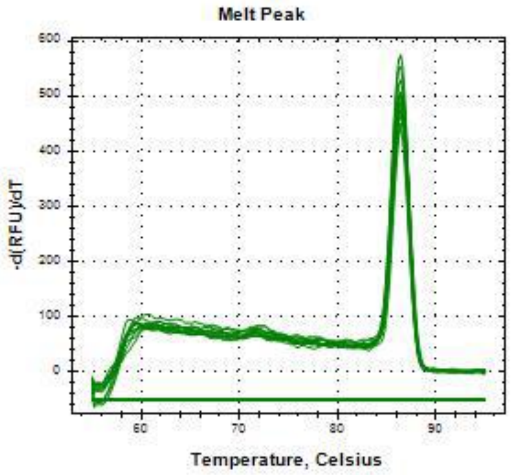

Figure S6

a. Brain

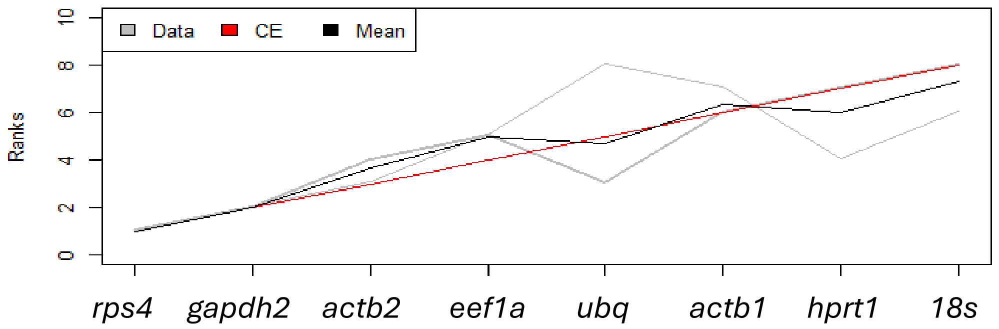

b. Gut

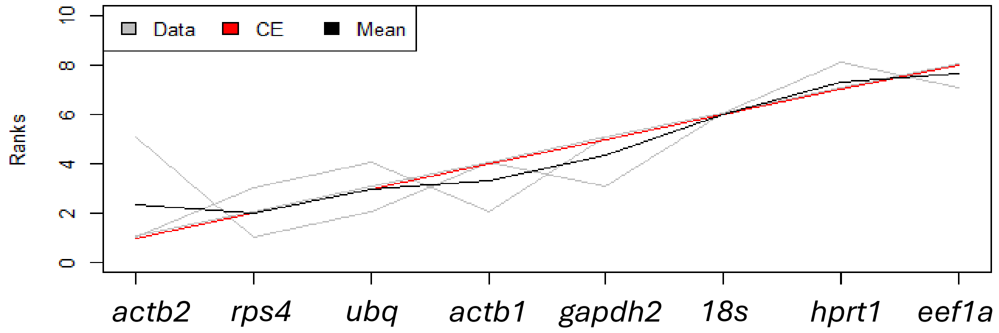

c. Kidney

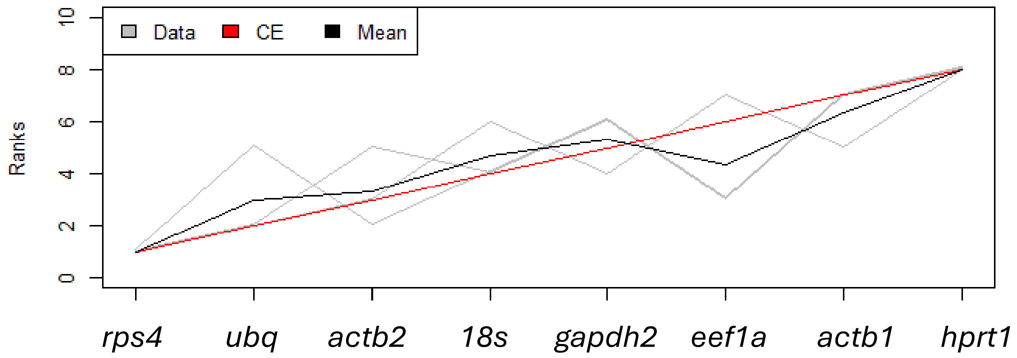

Figure S6. Integrated stability ranking by RankAggreg, using the data obtained from GeNorm, NormFinder and RefFinder algorithms, for brain (**a**), gut (**b**) and kidney (**c**). Black line: mean rank position of each gene. Red line: consensus ranking (CE) generated by Monte Carlo algorithm

Figure S7

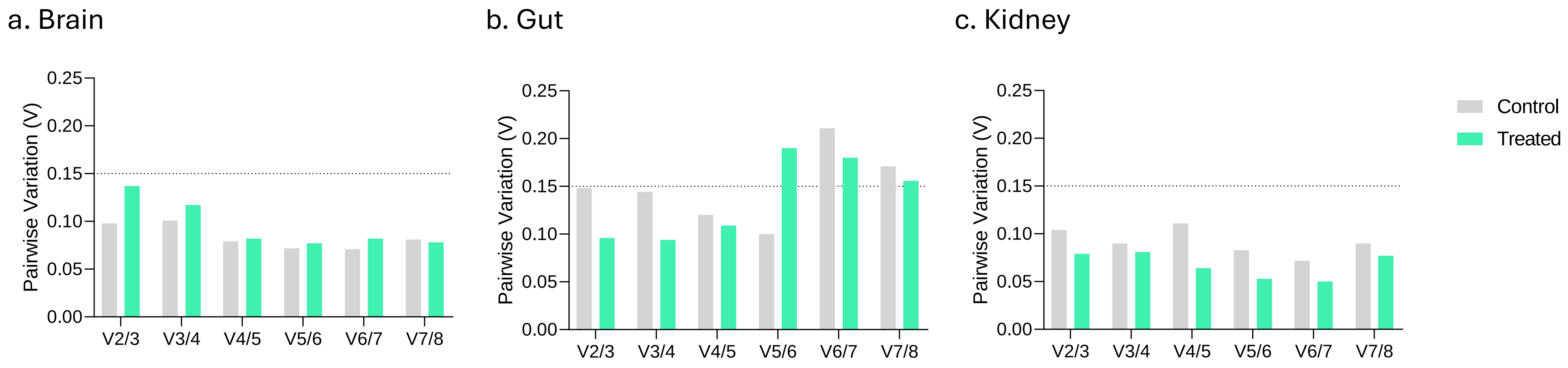

Figure S7. Pairwise Variation (V) results given by GeNorm in brain (**a**), gut (**b**) and kidney (**c**). The V-values were calculated using the normalisation factors NFn and NFn+1. Line at Y = 0.15 represents the cut-off value.
